# VeloPro: A pipeline integrating Ribo‐seq and AlphaFold deciphers association patterns between translation velocity and protein structure features

**DOI:** 10.1002/imt2.148

**Published:** 2023-11-19

**Authors:** Bian Bian, Toshitaka Kumagai, Yutaka Saito

**Affiliations:** ^1^ Department of Computational Biology and Medical Sciences Graduate School of Frontier Sciences, The University of Tokyo Kashiwa Japan; ^2^ Artificial Intelligence Research Center National Institute of Advanced Industrial Science and Technology (AIST) Koto‐ku Japan; ^3^ Fermlab Inc. Koto‐ku Japan; ^4^ AIST‐Waseda University Computational Bio Big‐Data Open Innovation Laboratory (CBBD‐OIL) Shinjuku‐ku Japan; ^5^ Department of Data Science, School of Frontier Engineering Kitasato University Sagamihara Japan

## Abstract

VeloPro integrates Ribo‐seq data and AlphaFold2‐predicted 3D protein structure information for characterization of the association patterns between translation velocity and many protein structure features in prokaryotic and eukaryotic organisms across different taxonomical clades such as bacteria, fungi, protozoa, nematode, plants, insect, and mammals. We illustrated that association patterns between translation velocity and protein structure features differ across organisms, partially reflecting their taxonomical relationship.

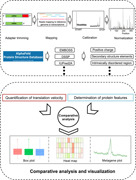

## INTRODUCTION

Translation is an essential process for protein biogenesis, and translation velocity (also known as ribosome dwell times) plays a crucial role in modulating protein co‐translational folding [1]. The protein co‐translational folding process starts when a nascent peptide chain emerges within the ribosome exit tunnel [2]. Translation velocity variation may modulate protein folding by enabling the formation of structural intermediates within the ribosome exit tunnel [2, 3]. Moreover, the co‐translational folding is facilitated by the electrostatic interactions between the proteins and the vestibule of the ribosomal tunnel [4]. Many studies have shown that larger proteins with more sophisticated structural properties may rely more on co‐translational folding to avoid misfolding and aggregation, which further illustrates the paramount importance of co‐translational folding [5]. Ribosomes actively engage in the folding of knotted proteins by threading nascent chains, which emerge from ribosome exit channels, through loops created by an earlier synthesized segment of the same protein; thus, co‐translational folding processes can be necessary to achieve the native structure of proteins whose polypeptide chain is deeply knotted [6, 7].

With rapid development in deep sequencing technologies, a revolutionary approach, ribosome profiling (also called Ribo‐seq) has been utilized to probe and quantify a local translation velocity at the codon resolution level in vivo [8]. As Ribo‐seq footprint density is considered to negatively correlate with the local translation velocity, Ribo‐seq has been successfully utilized to understand local translation velocity in bacteria and eukaryotes [9, 10, 11, 12].

Translation velocity is known to be associated with mRNA sequence features [13, 14, 15, 16]. Many pioneering studies illustrated that Shine–Dalgarno (SD)‐like sequences strongly impede translation [17] while some research argues that SD‐like motifs have little effect on translation elongation [18]. Codon usage bias is another critical factor strongly affecting translation kinetics [19]. Codon usage bias is considered to correlate with tRNA concentration in vivo and rare codons cause ribosome stalling on an mRNA during translation [1, 11]. Furthermore, mRNA secondary structure influences translation velocity, as the mRNA hairpin or pseudoknot structure could hinder ribosome movement during translation [20, 21]. More recently, Tajima et al. found that the relationship between translation efficiency and mRNA sequence features differs across nine microorganisms partially reflecting their taxonomy [13].

Despite the accumulating studies on mRNA sequence features, few studies have investigated the association between protein features and translation velocity [22, 23]. Zhou et al. observed that RNA regions coding for alpha‐helix or beta‐sheet preferentially use frequent codons, while those coding for coil regions prefer to use less frequent codons or rare codons in the filamentous fungus *Neurospora* [24]. A previous study also indicated that translation velocity is different between RNA regions coding for coil and structured regions; it becomes slower in mRNA sequences coding for coil regions than those coding for alpha‐helix or beta‐strand regions in yeast and humans [23], while they raised codon usage bias as a factor to explain these differences. Moreover, previous research has shown that positively charged amino acid is the primary inhibitor of translation velocity in yeast [22] and could also lead to ribosome stalling in Arabidopsis chloroplast [25]. Another study suggested that some short nascent peptide sequences could be associated with ribosome stalling in yeast, such as PPP and LKK amino acid sequences [26]. However, the previous studies have focused on a few specific organisms, mostly yeast and humans, raising the question that to what extent these association patterns apply to diverse organisms (Supporting Information S2: Table S1). Revealing the association between translation velocity and protein features in a broader range of taxonomy will help us better understand factors that may affect co‐translational folding.

In this study, we developed VeloPro, a pipeline that integrates publicly available ribosome profiling data and AlphaFold2‐predicted 3D protein structure information to investigate the association pattern between translation velocity and protein structure features in 12 organisms (Supporting Information S2: Table S2) across different taxonomical clades, including bacteria (*Escherichia coli* and *Pseudomonas aeruginosa*), fungi (*Saccharomyces cerevisiae* and *Candida albicans*), protozoa (*Trypanosoma brucei*), nematode (*Caenorhabditis elegans*), plants (*Arabidopsis* and maize), insect (fruit fly), and mammals (human, rat, and mouse). For each organism, we analyze more protein features than in previous studies, including protein secondary structure elements, proline residue, positively charged amino acid, relatively accessible surface area (rASA), intrinsically disordered region (IDR) scores, and local contact order. We revealed that the association pattern between translation velocity and protein structure features differs across organisms, partially reflecting their taxonomical relationship. We found that the association between translation velocity and positively charged amino acids previously reported in yeast did not apply to prokaryotes. We also found that the association patterns for secondary structure, rASA, and local absolute contact order are widely conserved in most organisms.

## RESULTS

### Workflow of VeloPro

**Figure 1:**
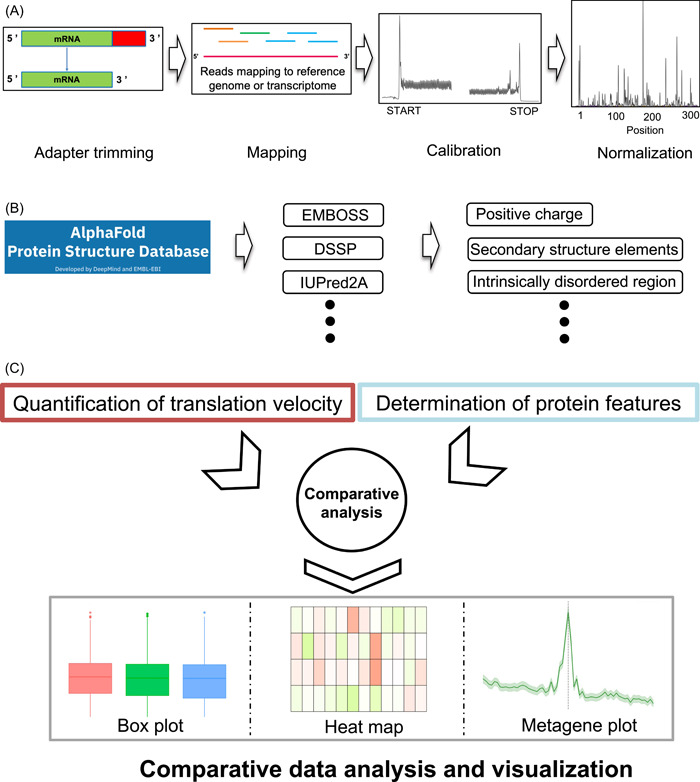
Workflow of VeloPro. (A) Pipeline for translation velocity quantification including adapter trimming, mapping, calibration, and normalization. (B) Pipeline for protein structure features determination from AlphaFold. (C) Schematic of the comparative data analysis and visualization between translation velocity and protein structure features used in this study.

In this study, we developed a pipeline VeloPro by combining ribosome profiling and AlphaFold‐predicted protein 3D structure data and then investigated the association pattern between translation velocity and protein structure features. The workflow is described in Figure 1.

For ribosome profiling analysis, previous reports indicate that Ribo‐seq results are influenced by many factors, including growth conditions, cell or tissue heterogeneity, protocol‐specific biases, sequencing coverage depth and biases, experimental artifacts, and unknown organism‐specific inherent factors [27]. Moreover, previous research reported that some bioinformatic bias may also lead to conflicting conclusions [12]. Therefore, in our analysis, we adopted the unified bioinformatic analysis pipeline for all the Ribo‐seq data analyses and protein feature determination in all the organisms. Moreover, for the quantification of translation velocity through Ribo‐seq data analysis, we used stringent offset identification and calibration (Figure 1A).

In our workflow, we first checked the quality of FASTQ files by FastQC (Supporting Information S2: Table S3) and then performed read mapping. The resultant BAM files were subsequently fed into the calibration process to obtain the scaled footprints that were considered inversely correlated with the translation velocity (Figure 1A). Then, protein‐predicted 3D structure information from the AlphaFold database was used to determine the protein structure features including protein secondary structure elements, rASA, IDR scores, and local contact order (Figure 1B). Next, we performed the comparative analysis between translation velocity and protein structure features, including rASA, IDR scores, and local contact order, and metagene analysis for proline residue and positively charged amino acids (Figure 1C).

### Quantification of translation velocity by Ribo‐seq analysis in diverse organisms

To quantify the translation velocity for the association and metagene analysis, we analyzed publicly available Ribo‐seq data sets (Supporting Information S2: Table S2). We selected 12 organisms in diverse taxonomical clades, including bacteria, protozoa, fungi, nematodes, insects, plants, and mammals. Depending on the organisms, 15–604 million reads were uniquely mapped to the reference sequences (Supporting Information S2: Tables S4 and S5). The distribution of the lengths of mapped reads varied in each organism (Supporting Information S1: Figure S1, Supporting Information S2: Table S4). To calibrate the position of RPF to the ribosomal P site in each organism, we used the MiMB_ribosome_profiling pipeline [28]. For each data set, we extracted reads whose lengths were most abundant in Supporting Information S1: Figure S1. Then, the offset for each read length was determined according to the manual's guidance. The determined offset values are summarized in Supporting Information S2: Table S4. After calibration, the coverage plots were generated around the start codon and stop codon, which exhibited three‐nucleotide periodicity within the coding region in all the organisms (Supporting Information S1: Figure S2). Such three‐nucleotide periodicity has been considered an indication of good data quality for performing codon‐resolution analysis of Ribo‐seq data [28]. We used this coverage information to calculate the scaled footprints as a measure of translation velocity at each codon position (Supplementary materials and methods). Fewer footprints were considered to indicate higher translation velocity, while more footprints were considered to indicate low translation velocity or ribosomal pausing.

### Comparison of translation velocity between different secondary structure elements in diverse organisms

Proteins could be folded during the translation process, which is called the co‐translational folding process [29]. Some rare codons have been associated with co‐translational folding intermediates of a single domain or a secondary structure within a protein, suggesting pauses during protein synthesis could facilitate co‐translational folding, thereby promoting the formation of its native structures [30]. Here, we compared the translation velocity between different protein secondary structure elements to investigate their associations.

**Figure 2:**
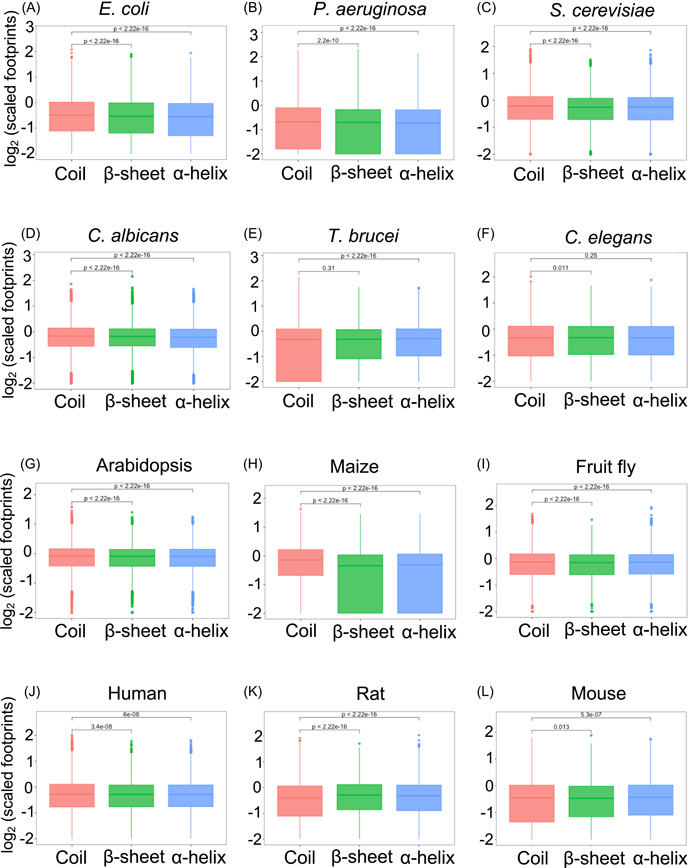
Comparisons of scaled footprints between the structured (i.e., α‐helix and β‐sheet) and coil regions in 12 organisms. *p*‐Values were calculated by two‐sided Wilcoxon rank‐sum test.

In both prokaryotes and most eukaryotes, the translation velocity in RNA sequence coding for coil regions was significantly slower than that coding for alpha‐helix and beta‐sheet regions (Figure 2), suggesting that ribosomes tend to stall in the RNA sequence coding for the unstructured regions than that coding for the structured regions. A previous study reported a higher stalling probability in RNA sequence coding for unstructured regions than that coding for structured regions, while the analysis was limited in yeast and humans [23]. We revealed that this pattern is consistent across broader organisms. Unexpectedly, the pattern in rat and *T. brucei* was the opposite; RNA sequence coding for coil regions exhibited faster translation velocity than that coding for alpha‐helix and beta‐sheet regions (Figure 2E,K).

### The effect of proline residue on translation velocity is widely conserved

The previous report has illustrated that proline amino acid acts as both a poor A‐site acceptor and peptidyl donor during the peptide bond formation process [31] and proline could slow down peptide bond formation in the translation process [32]. Many previous studies have indicated that polyproline stretches within an amino acid sequence could induce ribosome pausing in both prokaryotes and eukaryotes [26, 31]. However, few studies have analyzed the effect of proline residue on translation velocity in a single amino acid resolution.

In our metagene analysis for proline residue, we observed a strong peak around proline residue in yeast and humans suggesting that proline‐caused stalling phenomenon could be successfully detected by our strategy (Supporting Information S1: Figure S3C,J), which is consistent with the previous studies that proline residue leads to ribosome stalling events in human and yeast. In addition, we also observed one or more peaks around proline residue in many eukaryotes, such as *C. albicans*, *Arabidopsis*, maize, and fruit fly (Supporting Information S1: Figure S3). Intriguingly, we did not observe a peak around the proline residue in prokaryotes such as *E. coli* and *P. aeruginosa* (Supporting Information S1: Figure S3A,B) although previous research claimed that the proline–proline motif could cause ribosome stalling in bacteria [33].

### Positively charged amino acids may impose a different effect on translation velocity between prokaryotes and eukaryotes

In a previous study, positively charged amino acids in a nascent peptide chain have been shown to strongly inhibit translation elongation speed in yeast [22].

Our metagene analysis in yeast agreed with the previous research (Supporting Information S1: Figure S4C), showing a strong positive peak around the site of positively charged amino acids. In addition, we observed similar positive peaks around positively charged amino acids in most eukaryotes such as *T. brucei*, *C. albicans*, fruit fly, *Arabidopsis*, maize, and humans (Supporting Information S1: Figure S4), suggesting that translation pausing by positively charged amino acids is widely conserved across eukaryotes. For prokaryotes, there are no previous reports regarding the effect of positively charged amino acids on translation velocity. We found that the positively charged amino acids exhibited a different manner in prokaryotes compared to that in eukaryotes; they indicated negative peaks in the metagene plot (Supporting Information S1: Figure S4A,B).

### Correlation analysis between translation velocity and codon usage frequency among diverse organisms

Codon usage has been shown to affect local translation velocity [12]. Rare or nonoptimal codons could slow down the translation velocity, providing sufficient time for protein structures to fold correctly [34], thus fine‐tuning the co‐translational folding process [3, 12].

**Figure 3:**
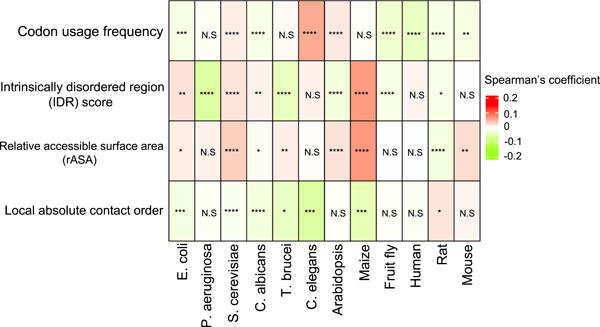
Heatmap of partial Spearman correlation coefficients between scaled footprints and protein structure features in 12 organisms. The mean of the partial correlation coefficients of all the analyzed genes is shown. Bonferroni‐corrected *p*‐value for multiple testing was calculated by one sample *t*‐test for the deviation of the mean from zero: *p < 0.05, **p < 2.2e−5, ***p < 2.2e−10, ****p < 2.2e−15. N.S, not significant. The raw values of the means and the Bonferroni‐corrected *p*‐values are shown in Supporting Information S2: Table S6.

Our partial correlation analysis in *E. coli*, *C. albicans*, fruit flies, humans, rats, and mice exhibited a significant negative correlation between the scaled footprints and codon usage frequency (Figure 3; Supporting Information S2: Table S6), which was in agreement with the previous reports. However, we also observed an opposite result, that is, a significant positive correlation between codon usage frequency and scaled footprints in *S. cerevisiae*, *C. elegans*, and *Arabidopsis* (Figure 3; Supporting Information S2: Table S6). These results indicate that codon usage alone does not explain all the variations in translation velocity, suggesting the contribution of other unknown factors. To explore these factors, we performed the association analysis between protein features and translation velocity as described in the next sections.

### Divergent association pattern between translation velocity and IDR scores in diverse organisms

IDR is defined as sequence segments that do not form specific 3D structures. They are often characterized by a high frequency of polar or charged amino acids with the lack of enough hydrophobic amino acids to enable cooperative folding [35, 36]. IDR plays a vital role in mediating interactions with other molecules by serving as the platform or scaffold [36, 37, 38]. In a previous study, the relationship between IDR and translation was investigated using the codon adaptation index (CAI) as a surrogate of the translation elongation rate [24]. They observed a significant negative correlation between CAI and protein disorder tendency in *E. coli*, *S. cerevisiae*, and *C. elegans* [24]. However, no study has shown direct evidence based on translation velocity measured by Ribo‐seq. Since our analysis showed the difference in translation velocity depending on secondary structures (Figure 2), we also explored whether IDR is associated with translation velocity.

In our partial correlation analysis, we observed a significant positive correlation between the scaled footprints and IDR scores in *E. coli* and *S. cerevisiae* (Figure 3; Supporting Information S2: Table S6), and this pattern was reminiscent of the previous CAI‐based findings [24]. However, in the bacteria clade, we also observed an opposite association pattern in *P. aeruginosa* compared to that in *E. coli*. In the fungi clade, both *S. cerevisiae* and *C. albicans* exhibited a positive correlation between scaled footprints and IDR scores. In plant clades, we observed opposite patterns between Arabidopsis (negative correlation) and maize (positive correlation).

In addition, we also explored the correlation between translation velocity and IDR score in different regions of proteins, including the N‐terminal, middle, and C‐terminal segments (Supporting Information S1: Figure S5; Supporting Information S2: Table S7). In the N‐terminal region, the correlation was overall weak, while a significantly positive correlation was detected for a few organisms. In the C‐terminal region, *S. cerevisiae* and *C. albicans* exhibited a significantly positive correlation, while higher eukaryotes such as *Arabidopsis*, fruit flies, and mice showed a significantly negative correlation. The correlation pattern in the middle region remained consistent with the analysis using the entire protein region.

In summary, the association between IDR scores and translation velocity was divergent among different organisms. This may reflect their evolutionary divergence while we could not make deeper interpretations.

### Conserved association pattern of rASA and absolute contact order with translation velocity

The rASA is a measure of the solvent molecule contact of an amino acid residue in a protein 3D structure [39, 40]. Based on the rASA value, the interface of protein could be classified into core, rim, and support. Lower rASA indicates a buried residue inside a protein structure (core), while higher rASA indicates a residue exposed to solvent (rim) [39].

In our partial correlation analysis, most of the organisms exhibited a positive correlation with a significant *p*‐value between scaled footprints and rASA (Figure 3; Supporting Information S2: Table S6), suggesting that RNA sequence coding for buried regions is preferentially translated faster than that coding for the exposed region. An exception was the rat, which showed a negative correlation, being opposite to the other organisms.

Contact order is a measure of the locality of amino acid contacts in a protein 3D structure [41]. A larger value of contact order indicates that amino acids tend to get in contact with distant amino acids with large separations in a sequence. Contact order has been used to compare the topological difference and shown to correlate with many protein properties, such as protein folding rates and transition state placements [41, 42].

In our partial correlation analysis, we found that the scaled footprints are significantly negatively correlated with local absolute contact order in *E. coli* (Figure 3; Supporting Information S2: Table S6). This is consistent with the previous report that protein substructures with lower contact orders are associated with conserved rare codons in *E. coli* [43]. Furthermore, we found a significantly negative correlation between scaled footprints and local absolute contact order in most of the organisms except rats (Figure 3; Supporting Information S2: Table S6).

## DISCUSSION

Translation velocity plays an essential role in regulating protein abundance and ensuring the protein's functional integrity through co‐translational folding [44]. Previous reports have indicated that translation velocity is not uniform along an mRNA sequence and tried to find sequence features determining these variations [1, 25]. However, most previous studies focused on the association between mRNA sequence features and translation velocity in specific model organisms. Recently, Tajima et al. [13] discovered the diversity and commonalities of mRNA sequence determinants for protein expression across nine microorganisms. However, they did not explore the associated protein structure features. The recent revolution in the prediction of protein 3D structure enables the proteome‐wide investigation of structural information in diverse organisms [45, 46]. In our study, by combining Ribo‐seq and AlphaFold‐predicted protein 3D structure data, we revealed many protein structure features associated with translation velocity. By analyzing the 12 organisms across various evolutionary clades, we also identified the features showing the conserved association pattern among many organisms while revealing several features with divergent association patterns. Thus, our study provides a comprehensive view of protein features correlated with translation velocity, which may affect the co‐translational folding process across the clades.

Overall, translation velocity patterns in mRNA sequence coding for structured (alpha‐helix and beta‐sheet) regions and disorder (coil) regions were widely observed in both prokaryotes and eukaryotes, which may reflect the evolutionary conservation and the same evolutionary pressure. This pattern suggests that translation velocity becoming slower in the mRNA sequence coding for disordered regions could ensure sufficient time for correct co‐translational folding in structured regions or domains. This finding is consistent with previous reports that the ribosome pausing sites located at protein domain boundaries facilitate and favor co‐translational folding, thus avoiding the potential misfolding [47].

In addition, the association pattern between rASA and translation velocity was also conserved in both prokaryotes and eukaryotes, suggesting that RNA sequence coding for exposed regions tends to be translated slower than that coding for buried regions in all the tested organisms except rats. In addition, this association pattern was also observed between translation velocity and local absolute contact order.

The association pattern in rats was different from the other mammals in many cases; for example, it showed lower‐scaled footprints in coil regions compared to alpha‐helix and beta‐sheet regions, while it was the opposite in the other mammals (Figure 2J–L). To explore whether these exceptional results are contingent on the data set, we conducted an additional analysis using another data set derived from rat brain, mouse kidney, *E. coli*, and human hela cell, respectively (Supporting Information S2: Table S2). Notably, the two data sets chosen for each organism originate from different research groups. Intriguingly, the association patterns for each organism were similar between the two data sets for most of the protein features including protein secondary structure, proline residue, positive charge, IDR scores, and local absolute contact order (Supporting Information S1: Figures S6–S10). These results suggest that the extraordinary pattern in rats may not be caused by data set‐specific artifacts, but probably due to unknown inherent factors. A possible explanation is the more tissue‐specific nature of gene expression in higher eukaryotes. Our integrative analysis of Ribo‐seq and protein features requires genes with high read coverages (>60%); thus, the number of analyzed genes was relatively small in higher eukaryotes (Supporting Information S2: Table S4), which might affect the results in each data set.

Positively charged amino acids could strongly arrest ribosome movement, and this influence cannot be compensated by the mRNA structure and codon usage bias in yeast [22]. Moreover, a positive charge also induces ribosome pausing in the Arabidopsis chloroplast [25]. The most likely explanation is that positively charged residues in the nascent peptide chain could interact with the negatively charged exit tunnel walls, and these electrostatic interactions retard translation [22, 48]. In our analysis, we indeed observed a similar pattern in yeast and Arabidopsis; there were several peaks of scaled footprints around positively charged amino acids in yeast and one peak in *Arabidopsis*, suggesting that positively charged amino acids inhibit translation in yeast and Arabidopsis. On the contrary, our results suggested that positive charges may speed up the translation in prokaryotes such as *E. coli* and *P. aerugisona* (Supporting Information S1: Figure S4A,B). The possible explanation is that there is an evolutionary divergence between prokaryotes and eukaryotes in the geometry of the exit tunnel, which is induced by constricted regions (troughs in the exit tunnel) that determine the electrostatic potential [49]. This variation in electrostatic potential may lead to different association patterns between positively charged amino acid residues and translation velocity in diverse organisms.

Proline tends to cause ribosome pausing in both prokaryotes and eukaryotes, as the amino side chain of proline is known to act as both a poor A‐site acceptor and a poor donor in the P‐site in the peptidyl transfer reaction [50]. This is likely due to its restricted conformational flexibility, which impedes translational elongation, as reported in *S. cerevisiae*, *Saccharomyces paradoxus*, and zebrafish [27]. In addition, the effect of proline on ribosome stalling could be additive [23]; the translational stalling becomes strong at di‐proline motifs (XPPX) and polyproline stretches (XPPPX). However, the ribosome stalling at polyproline motifs could be rescued by the translation elongation factor EF‐P in bacteria [51], or initiation factor IF5A in eukaryotes [31, 52] and loss of function of EF‐P will lead to strong ribosome pausing at proline‐rich motifs in *E. coli* [53]. Both EP‐P and eIF5A could enhance the peptidyl transferase activity of the ribosome and facilitate the reactivity of poor substrates such as proline [52]. In our result (Supporting Information S1: Figure S3), the effect of the proline residue in prokaryotes and eukaryotes was different, possibly due to the protein function diversity in resolving proline‐induced ribosome stalling (EF‐P in bacteria and IF5A in eukaryotes).

In this study, we solely explored the relationship between translation velocity and protein structure features. Nonetheless, other relevant studies have indicated that the rate of protein folding is likewise correlated with protein structures such as protein size, shape, packing, and cross‐sectional radius [54, 55, 56]. Additionally, it is noteworthy that proteins exhibiting simple folding kinetics tend to fold more rapidly in bacterial systems compared to eukaryotic systems, which gives hints regarding the evolutionary divergence in protein structures [57].

## CONCLUSION

In summary, we performed the association analysis between translation velocity and various protein features based on publicly available Ribo‐seq data. We revealed the conserved relationship between translation velocity and protein structure features (secondary structure elements, rASA, local absolute contact order), suggesting that organisms impose different translation velocities on protein domains to ensure correct protein folding. Our study could also provide insights into mRNA sequence design to enhance recombinant protein expression. Previous studies have only considered mRNA sequence features such as codon usage and mRNA secondary structures to design mRNA. Our study shows that protein structures are also associated with translation velocity, suggesting the possibility that protein structure features can also be incorporated to design mRNA to fine‐tune co‐translation folding. Our findings could contribute to recombinant protein expression in biomanufacturing fields.

## AUTHOR CONTRIBUTIONS

Bian Bian and Yutaka Saito conceived this study and designed the method. Bian Bian, Toshitaka Kumagai, and Yutaka Saito wrote the codes. Bian Bian and Yutaka Saito analyzed and interpreted the data. Bian Bian and Yutaka Saito wrote and revised the manuscript. Yutaka Saito supervised this project. All authors have read the final manuscript and approved it for publication.

## CONFLICT OF INTEREST STATEMENT

The authors declare no conflict of interest.

## Supporting information

Supporting_information.

Supporting_information.

## Data Availability

All data generated or analyzed in this study are included in this manuscript and Supporting Information. VeloPro pipeline is publicly available on GitHub (https://github.com/ytksailab-org/VeloPro). Supplementary materials (methods, figures, tables, scripts, graphical abstract, slides, videos, Chinese translated version, and updated materials) may be found in the online DOI or iMeta Science (http://www.imeta.science/).
