# Supplementary material for: VeloPro: A pipeline integrating Ribo‐seq and AlphaFold deciphers association patterns between translation velocity and protein structure features
Source: Imeta. 2023 Nov 19;2(4):e148. doi: 10.1002/imt2.148 (PMC10989810; doi:10.1002/imt2.148)
Supplement: Supplementary file 1 — Supporting_information. [file IMT2-2-e148-s001.docx]

Supplementary Information to:

**VeloPro: A pipeline integrating Ribo-seq and AlphaFold deciphers association patterns between translation velocity and protein structure features**

Running title: VeloPro deciphers translation velocity associated protein features

Bian Bian ^1,2^, Toshitaka Kumagai ^3^, Yutaka Saito ^1,2,4,5*^

1. Department of Computational Biology and Medical Sciences, Graduate School of Frontier Sciences, The University of Tokyo, Kashiwa, Chiba 277-8561, Japan.

2. Artificial Intelligence Research Center, National Institute of Advanced Industrial Science and Technology (AIST), Koto-ku, Tokyo 135-0064, Japan.

3. Fermlab Inc., Koto-ku, Tokyo 135-0021, Japan.

4. AIST-Waseda University Computational Bio Big-Data Open Innovation Laboratory (CBBD-OIL), Shinjuku-ku, Tokyo 169-8555, Japan.

5. Department of Data Science, School of Frontier Engineering, Kitasato University, 1-15-1 Kitazato, Minami-ku, Sagamihara, Kanagawa 252-0373, Japan.

*Correspondence: yutaka.saito@aist.go.jp (Yutaka Saito)

**Supplementary materials and methods**

**Ribosome profiling data processing, calibration, and normalization**

Publicly available ribosome profiling (Ribo-seq) datasets were collected from NCBI for 12 organisms (Table S2) which comprise diverse taxonomical clades including bacteria (*E. coli* and *P. aeruginosa*) [1, 2], fungi (*S. cerevisiae* and *C. albicans*) [3, 4], protozoa (*T. brucei*) [5], nematode (*C. elegans*) [6], plants (Arabidopsis and maize) [7, 8], insect (fruit fly) [9], and mammals (human, rat, and mouse) [10-12]. We first checked the quality of sequencing data with FastQC version 0.11.2 (https://www.bioinformatics.babraham.ac.uk/projects/fastqc/). Then the Ribo-seq raw reads were trimmed by the cutadapt version 3.5 tool [13] to remove the universal adapter according to the original paper’s instructions; Alternatively, we provide the option to use fastp [14] for quality check and adapter removal in our pipeline. We downloaded the reference genome sequences in FASTA format and annotations in GFF format for each organism from NCBI (https://www.ncbi.nlm.nih.gov/genome/), then generated the reference transcriptome by gffread version 0.12.1 for eukaryotes [15]. The detailed genome version information of each organism is shown in Table S5. The trimmed raw reads were aligned to each organism's reference transcriptome (for bacteria, we used the genome mapping) by Bowtie version 1.0.0 [16] with the following parameters: bowtie -v 2 -m 1, which only allows uniquely mapped reads with no more than 2 mismatches. The resultant sorted BAM format files were subsequently processed by MiMB_ribosome_profiling (https://github.com/AlexanderBartholomaeus/MiMB_ribosome_profiling) in R (version 3.5.2) to determine the offset and then to calibrate the positions of ribosome protected footprints (RPF) [17, 18]. Subsequently, the calibrated BAM format file was processed to generate the nucleotide-wise RPF counts. Then, the resultant nucleotide-wise RPF counts were converted into codon-wise read counts by summing read counts at 3 nucleotides in each codon. To normalize the codon-wise read counts, they were divided by the average of all codons in each transcript. These values were referred to as "scaled footprints" where a value > 1 (or < 1) was considered to indicate decreased (or increased) translation velocity at the codon. Similar normalization procedures were also used in previous studies [19, 20]. In addition, the genes with a sequence coverage rate of less than 60% were discarded in each organism. All the adapter trimming, mapping, offset calibration stats, and the number of genes used were summarized in Table S4.

**Determination of protein structure features**

To calculate the protein structure features, PDB format files containing the amino acid sequence and predicted protein 3D structure information of each organism were downloaded from AlphaFold Protein Structure Database, EMBL-EBI (https://alphafold.ebi.ac.uk/download, last accessed: March, 2023) [21, 22].

Protein secondary structure elements and rASA were calculated by using DSSP tools [23, 24] in biopython [25] with Bio.PDB.DSSP module (Version 2.1.0) under Python 3.7.2 with the default setting, by which each amino acid residue in a protein was assigned with either of the eight types of protein secondary structure elements (coil, α-helix, β-sheet, β-bridge, 3-helix, 5 helix, hydrogen-bonded turn, and bend) and the rASA value [26].

Relative contact order (CO) is a measure of the sequence separation of contacting amino acids within a folded protein [27] defined by the following mathematical formula: $CO=\frac{1}{N\cdot L}\sum_{1}^{N} \Delta S_{i,j}$ where N is the total number of contacts, $\Delta S_{i,j}$ denotes the sequence separation between contacting residues *i* and *j*, and *L* is the length of the protein. A previous study showed that absolute contact order is a more appropriate indicator to estimate protein folding state kinetics [28], which is defined as *Abs_CO* = *CO* $\times$ *L*. The original versions of relative and absolute contact orders are defined for an entire protein, not for each amino acid residue. To evaluate their associations with Ribo-seq footprints at each amino acid residue, we defined the modified versions named "local" relative and absolute contact orders. Specifically, we computed both relative and absolute contact orders for every sequence segment from 1 to residue *i*, which are defined as local relative and absolute contact orders, respectively. We calculated these local contact orders by using a modified version of the original Perl script provided by [27]. We observed that local relative and absolute contact orders tended to have high values at the C-termini of proteins regardless of their 3D structures. Thus, we also tested "normalized" local contact orders, where we divided local relative contact order at each residue *I* by its average across all proteins in the organism.

To identify disordered protein regions, IUPred2A [29] was used to estimate the intrinsically disordered region (IDR) score at each amino acid residue with the option "long" under Python 3.7.2. In addition, to investigate the correlation between translation velocity and IDR score in different regions of proteins, we define the N-terminal region as the first 10 amino acids from the protein's N-terminus, the C-terminal region as the last 10 amino acids from the protein's C-terminus, and the remaining segment as the middle part of the protein.

Additionally, for comparison with an mRNA feature, we used the feature of codon usage frequency in each organism, which was downloaded from CoCoPUTs databases (https://dnahive.fda.gov/dna.cgi?cmd=codon_usage&id=537&mode=cocoputs, last accessed: December, 2022) [30].

**Metagene analysis for proline residue and positively charged amino acid**

Proline residue and positively charged amino acid information were determined by using the pepinfo tool in the EMBOSS package (Version: 6.5.7.0) [31]. For metagene analysis, sequence regions around the amino acid residues of interest (proline or positively charged amino acids) were extracted with the window size of 61 (from - 30 to + 30 around the amino acids). The scaled footprints in these regions were averaged across the corresponding positions in the window and then the means and 95% confidence intervals were calculated.

**Statistical analysis**

The scaled footprints between different secondary structure elements were compared by the Wilcoxon rank-sum test. The Spearman rank partial correlation analysis between scaled footprints and protein features including rASA, IDR scores, local contact order, and codon usage frequency was conducted by Python. To avoid indirect association, partial correlation analysis was performed by pingouin module (v0.5.3) [32] in Python 3.7.2. We calculated the partial correlation of translation velocity and each feature with all the other features set as controlling variables. The partial correlation coefficient was calculated for each gene. The mean of partial correlation coefficients was reported with the statistical significance for the deviation from zero using one sample t-test with Bonferroni correction for multiple testing corrections. Wilcoxon rank-sum test and one sample t-test were performed by R. ComplexHeatmap package [33] in R was used to generate the heatmap plots.

**REFERENCES**

1. Mohammad, Fuad, Christopher J Woolstenhulme, Rachel Green, Allen R Buskirk. 2016. “Clarifying the Translational Pausing Landscape in Bacteria by Ribosome Profiling.” *Cell Rep* 14: 686-694. <https://doi.org/10.1016/j.celrep.2015.12.073>

2. Grady, Sarah L, Stephanie A.Malfatti, Thusitha S.Gunasekera, Brian K.Dalley, Matt G.Lyman, Richard C.Striebich, Michael B. Mayhew, Carol L. Zhou, Oscar N. Ruiz, Larry C. Dugan. 2017. “A comprehensive multi-omics approach uncovers adaptations for growth and survival of Pseudomonas aeruginosa on n-alkanes.” *BMC Genomics* 18: 334. <https://doi.org/10.1186/s12864-017-3708-4>

3. Pop, Cristina, Silvi Rouskin, Nicholas T Ingolia, Lu Han, Eric M Phizicky, Jonathan S Weissman, Daphne Koller. 2014. “Causal signals between codon bias, mRNA structure, and the efficiency of translation and elongation.” *Mol Syst Biol* 10: 770. <https://doi.org/10.15252/msb.20145524>

4. Muzzey, Dale, Gavin Sherlock, Jonathan S Weissman. 2014. “Extensive and coordinated control of allele-specific expression by both transcription and translation in Candida albicans.” *Genome Res* 24: 963-973. <https://doi.org/10.1101/gr.166322.113>

5. Jensen, Bryan C, Gowthaman Ramasamy, Elton J R Vasconcelos, Nicholas T Ingolia, Peter J Myler, Marilyn Parsons. 2014. “Extensive stage-regulation of translation revealed by ribosome profiling of Trypanosoma brucei.” *BMC Genomics* 15: 911. <https://doi.org/10.1186/1471-2164-15-911>

6. Stadler, Michael, Karen Artiles, Julia Pak, Andrew Fire. 2012. “Contributions of mRNA abundance, ribosome loading, and post- or peri-translational effects to temporal repression of C. elegans heterochronic miRNA targets.” *Genome Res* 22: 2418-2426. <https://doi.org/10.1101/gr.136515.111>

7. Liu, Ming-Jung, Szu-Hsien Wu, Jing-Feng Wu, Wen-Dar Lin, Yi-Chen Wu, Tsung-Ying Tsai, Huang-Lung Tsai, Shu-Hsing Wu. 2013. “Translational landscape of photomorphogenic Arabidopsis.” *Plant Cell* 25: 3699-3710. <https://doi.org/10.1105/tpc.113.114769>

8. Lei, Lei, Junpeng Shi, Jian Chen, Mei Zhang, Silong Sun, Shaojun Xie, Xiaojie Li, et al. 2015. “Ribosome profiling reveals dynamic translational landscape in maize seedlings under drought stress.” *Plant J* 84: 1206-1218. <https://doi.org/10.1111/tpj.13073>

9. Dunn, Joshua G, Catherine K Foo, Nicolette G Belletier, Elizabeth R Gavis, Jonathan S Weissman. 2013. “Ribosome profiling reveals pervasive and regulated stop codon readthrough in Drosophila melanogaster.” *eLife* 2: e01179. <https://doi.org/10.7554/eLife.01179>

10. Battle, Alexis, Zia Khan, Sidney H Wang, Amy Mitrano, Michael J Ford, Jonathan K Pritchard, Yoav Gilad. 2015. “Genomic variation. Impact of regulatory variation from RNA to protein.” *Science* 347: 664-667. <https://doi.org/10.1126/science.1260793>

11. Schafer, Sebastian, Eleonora Adami, Matthias Heinig, Katharina E. Costa Rodrigues, Franziska Kreuchwig, Jan Silhavy, Sebastiaan van Heesch, et al. 2015. “Translational regulation shapes the molecular landscape of complex disease phenotypes.” *Nat Commun* 6: 7200. <https://doi.org/10.1038/ncomms8200>

12. Wang, Hongwei, Yan Wang, Jiaqi Yang, Qian Zhao, Nan Tang, Congying Chen, Huihui Li, et al. 2021. “Tissue- and stage-specific landscape of the mouse translatome.” *Nucleic Acids Res* 49: 6165-6180. <https://doi.org/10.1093/nar/gkab482>

13. Martin, Marcel. 2011. “Cutadapt removes adapter sequences from high-throughput sequencing reads.” *EMBnet.journal* 17: 10-12. <https://doi.org/10.14806/ej.17.1.200>

14. Chen, Shifu. 2023. “Ultrafast one-pass FASTQ data preprocessing, quality control, and deduplication using fastp.” *iMeta* 2: e107. <https://doi.org/https://doi.org/10.1002/imt2.107>

15. Pertea, Geo, Mihaela Pertea. 2020. “GFF Utilities: GffRead and GffCompare [version 2; peer review: 3 approved].” *F1000Research* 9: <https://doi.org/10.12688/f1000research.23297.2>

16. Langmead, Ben, Cole Trapnell, Mihai Pop, Steven L Salzberg. 2009. “Ultrafast and memory-efficient alignment of short DNA sequences to the human genome.” *Genome Biol* 10: R25. <https://doi.org/10.1186/gb-2009-10-3-r25>

17. Bartholomaus, Alexander, Baban Kolte, Ayten Mustafayeva, Ingrid Goebel, Stephan Fuchs, Dirk Benndorf, Susanne Engelmann, Zoya Ignatova. 2021. “smORFer: a modular algorithm to detect small ORFs in prokaryotes.” *Nucleic Acids Res* 49: e89. <https://doi.org/10.1093/nar/gkab477>

18. Bartholomaus, Alexander, Zoya Ignatova. 2021. “Codon Resolution Analysis of Ribosome Profiling Data.” *Methods Mol Biol* 2252: 251-268. <https://doi.org/10.1007/978-1-0716-1150-0_12>

19. Zhang, Sai, Hailin Hu, Jingtian Zhou, Xuan He, Tao Jiang, Jianyang Zeng. 2017. “Analysis of Ribosome Stalling and Translation Elongation Dynamics by Deep Learning.” *Cell Syst* 5: 212-220 e216. <https://doi.org/10.1016/j.cels.2017.08.004>

20. Tunney, Robert, Nicholas J. McGlincy, Monica E. Graham, Nicki Naddaf, Lior Pachter, Liana F. Lareau. 2018. “Accurate design of translational output by a neural network model of ribosome distribution.” *Nature Structural & Molecular Biology* 25: 577-582. <https://doi.org/10.1038/s41594-018-0080-2>

21. Jumper, John, Richard Evans, Alexander Pritzel, Tim Green, Michael Figurnov, Olaf Ronneberger, Kathryn Tunyasuvunakool, et al. 2021. “Highly accurate protein structure prediction with AlphaFold.” *Nature* 596: 583-589. <https://doi.org/10.1038/s41586-021-03819-2>

22. Varadi, Mihaly, Stephen Anyango, Mandar Deshpande, Sreenath Nair, Cindy Natassia, Galabina Yordanova, David Yuan, et al. 2022. “AlphaFold Protein Structure Database: massively expanding the structural coverage of protein-sequence space with high-accuracy models.” *Nucleic Acids Res* 50: D439-D444. <https://doi.org/10.1093/nar/gkab1061>

23. Kabsch, Wolfgang, Christian Sander. 1983. “Dictionary of protein secondary structure: pattern recognition of hydrogen-bonded and geometrical features.” *Biopolymers* 22: 2577-2637. <https://doi.org/10.1002/bip.360221211>

24. Joosten, Robbie P, Tim A.H te Beek, Elmar Krieger, Maarten L Hekkelman, Rob W.W Hooft, Reinhard Schneider, Chris Sander, Gert Vriend. 2011. “A series of PDB related databases for everyday needs.” *Nucleic Acids Res* 39: D411-419. <https://doi.org/10.1093/nar/gkq1105>

25. Hamelryck, Thomas, Bernard Manderick. 2003. “PDB file parser and structure class implemented in Python.” *Bioinformatics* 19: 2308-2310. <https://doi.org/10.1093/bioinformatics/btg299>

26. Rost, Burkhard, Chris Sander. 1994. “Conservation and prediction of solvent accessibility in protein families.” *Proteins* 20: 216-226. <https://doi.org/10.1002/prot.340200303>

27. Plaxco, Kevin W, Kim T Simons, David Baker. 1998. “Contact order, transition state placement and the refolding rates of single domain proteins.” *J Mol Biol* 277: 985-994. <https://doi.org/10.1006/jmbi.1998.1645>

28. Ivankov, Dmitry N, Sergiy O Garbuzynskiy, Eric Alm, Kevin W Plaxco, David Baker, Alexei V Finkelstein. 2003. “Contact order revisited: influence of protein size on the folding rate.” *Protein Sci* 12: 2057-2062. <https://doi.org/10.1110/ps.0302503>

29. Mészáros, Bálint, Gábor Erdős, Zsuzsanna Dosztányi. 2018. “IUPred2A: context-dependent prediction of protein disorder as a function of redox state and protein binding.” *Nucleic Acids Research* 46: W329-W337. <https://doi.org/10.1093/nar/gky384>

30. Athey, John, Aikaterini Alexaki, Ekaterina Osipova, Alexandre Rostovtsev, Luis V Santana-Quintero, Upendra Katneni, Vahan Simonyan, Chava Kimchi-Sarfaty. 2017. “A new and updated resource for codon usage tables.” *BMC Bioinformatics* 18: 391. <https://doi.org/10.1186/s12859-017-1793-7>

31. Rice, Peter, Ian Longden, Alan Bleasby. 2000. “EMBOSS: the European Molecular Biology Open Software Suite.” *Trends Genet* 16: 276-277. <https://doi.org/10.1016/s0168-9525(00)02024-2>

32. Vallat, Raphael. 2018. “Pingouin: statistics in Python.” *Journal of Open Source Software* 3: <https://doi.org/10.21105/joss.01026>

33. Gu, Zuguang. 2022. “Complex heatmap visualization.” *iMeta* 1: e43. <https://doi.org/https://doi.org/10.1002/imt2.43>

**Supplementary figure and table legends**

**Figure S1** Distribution of read lengths of Ribo-seq dataset in 12 organisms.

**Figure S2** Coverage plots around start codon and stop codon by using calibrated reads in 12 organisms.

**Figure S3** Translation velocity around proline residue in 12 organisms. The vertical dashed line at X = 0 represents the proline residue. The Y-axis represents the average scaled footprints, Y = 1 indicates the baseline of translation velocity (average in each gene). Y > 1 indicates slower translation, while Y < 1 indicates faster translation. Ribbons represent the 95% confidence interval of the mean value.

**Figure S4** Translation velocity around positively charged amino acid in 12 organisms. The vertical dashed line at X = 0 represents the proline residue. The Y-axis represents the average scaled footprints, Y = 1 indicates the baseline of translation velocity (average in each gene). Y > 1 indicates slower translation, while Y < 1 indicates faster translation. Ribbons represent the 95% confidence interval of the mean value.

**Figure S5** Heatmap of non-partial spearman correlation coefficients between scaled footprints and intrinsically disordered region (IDR) score in N-terminal, Middle and C-terminal in 12 organisms. The mean of partial correlation coefficients of all the analyzed genes is shown. The P-value for multiple testing were calculated by one sample t-test for the deviation of the mean from zero: **p* < 0.05, ***p* < 2.2e-5, ****p* < 2.2e-10, *****p* < 2.2e-15. N.S, not significant. The raw values of the means and the P-values are shown in Table S7.

**Figure S6** Comparisons of scaled footprints between the structured (i.e., α-helix and β-sheet) and coil regions in additional dataset for rat, mouse, E. coli and human. P-value were calculated by two-sided Wilcoxon rank-sum test.

**Figure S7** Translation velocity around proline residue in additional dataset for rat, mouse, E. coli and human. The vertical dashed line at X = 0 represents the proline residue. The Y-axis represents the average scaled footprints, Y = 1 indicates the baseline of translation velocity (average in each gene). Y > 1 indicates slower translation, while Y < 1 indicates faster translation. Ribbons represent the 95% confidence interval of the mean value.

**Figure S8** Translation velocity around positively charged amino acid in additional dataset for rat, mouse, E. coli and human. The vertical dashed line at X = 0 represents the proline residue. The Y-axis represents the average scaled footprints, Y = 1 indicates the baseline of translation velocity (average in each gene). Y > 1 indicates slower translation, while Y < 1 indicates faster translation. Ribbons represent the 95% confidence interval of the mean value.

**Figure S9** Heatmap of partial spearman correlation coefficients between scaled footprints and 6 features of additional dataset in rat, mouse, *E. coli* and human. The mean of partial correlation coefficients of all the analyzed genes is shown. Bonferroni-corrected P-value for multiple testing were calculated by one sample t-test for the deviation of the mean from zero: **p* < 0.05, ***p* < 2.2e-5, ****p* < 2.2e-10, *****p* < 2.2e-15. N.S, not significant. The raw values of the means and the Bonferroni-corrected P-values are shown in Table S6.

**Figure S10** Heatmap of non-partial spearman correlation coefficients between scaled footprints and intrinsically disordered region (IDR) score in N-terminal, Middle and C-terminal in additional dataset for rat, mouse, *E. coli* and human. The mean of partial correlation coefficients of all the analyzed genes is shown. The P-value for multiple testing were calculated by one sample t-test for the deviation of the mean from zero: **p* < 0.05, ***p* < 2.2e-5, ****p* < 2.2e-10, *****p* < 2.2e-15. N.S, not significant. The raw values of the means and the P-values are shown in Table S7.

**Table S1** Summary of previous studies performing the association analysis between translation velocity and protein features.

**Table S2** Ribo-seq dataset information in 12 organisms used in this study. The accession numbers of NCBI SRA or GEO are shown.

**Table S3** FastQC report from quality check for fastq-format raw reads in 12 organisms. Qualified is represented by ✓, warning is represented by !, unqualified is represented by ✕, and not available is represented by NA.

**Table S4** Trimming, mapping and calibration stats in our analysis.

**Table S5** Reference genome version for mapping in 12 organisms.

**Table S6** Partial correlation between scaled footprints and protein structure features in 12 organisms with Bonferroni-corrected P-value.

**Table S7** Non-partial correlation between scaled footprints and intrinsically disordered region (IDR) score in protein different regions including N-terminal, middle and C-terminal in 12 organisms with P-value.

**Figure S1** Distribution of read lengths of Ribo-seq dataset in 12 organisms.

Figure S1. Distribution of read lengths of Ribo-seq dataset in 12 organisms.

**Figure S2** Coverage plots around start codon and stop codon by using calibrated reads in 12 organisms.

Figure S2. Coverage plots around start codon and stop codon by using calibrated reads in 12 organisms.

**Figure S3** Translation velocity around proline residue in 12 organisms. The vertical dashed line at X = 0 represents the proline residue. The Y-axis represents the average scaled footprints, Y = 1 indicates the baseline of translation velocity (average in each gene). Y > 1 indicates slower translation, while Y < 1 indicates faster translation. Ribbons represent the 95% confidence interval of the mean value.

Figure S3. Translation velocity around proline residue in 12 organisms. The vertical dashed line at X = 0 represents the proline residue. The Y-axis represents the average scaled footprints, Y = 1 indicates the baseline of translation velocity (average in each gene). Y > 1 indicates slower translation, while Y < 1 indicates faster translation. Ribbons represent the 95% confidence interval of the mean value.

**Figure S4** Translation velocity around positively charged amino acid in 12 organisms. The vertical dashed line at X = 0 represents the proline residue. The Y-axis represents the average scaled footprints, Y = 1 indicates the baseline of translation velocity (average in each gene). Y > 1 indicates slower translation, while Y < 1 indicates faster translation. Ribbons represent the 95% confidence interval of the mean value.

Figure S4. Translation velocity around positively charged amino acid in 12 organisms. The vertical dashed line at X = 0 represents the proline residue. The Y-axis represents the average scaled footprints, Y = 1 indicates the baseline of translation velocity (average in each gene). Y > 1 indicates slower translation, while Y < 1 indicates faster translation. Ribbons represent the 95% confidence interval of the mean value.

**Figure S5** Heatmap of non-partial spearman correlation coefficients between scaled footprints and (intrinsically disordered region) IDR score in N-terminal, Middle and C-terminal in 12 organisms. The mean of partial correlation coefficients of all the analyzed genes is shown. The P-value for multiple testing were calculated by one sample t-test for the deviation of the mean from zero: **p* < 0.05, ***p* < 2.2e-5, ****p* < 2.2e-10, *****p* < 2.2e-15. N.S, not significant. The raw values of the means and the P-values are shown in Table S7.

Figure S5. Heatmap of non-partial spearman correlation coefficients between scaled footprints and IDR score in N-terminal, Middle and C-terminal in 12 organisms. Statistical significance is indicated as follows: *p < 0.05, **p < 2.2e-5, **p < 2.2e-10, ****p < 2.2e-15.

**Figure S6** Comparisons of scaled footprints between the structured (i.e., α-helix and β-sheet) and coil regions in additional dataset for rat, mouse, *E. coli* and human. P-value were calculated by two-sided Wilcoxon rank-sum test.

Figure S6. Comparisons of scaled footprints between the structured (i.e., α-helix and β-sheet) and coil regions in additional dataset for rat, mouse, *E. coli* and human. P-value were calculated by two-sided Wilcoxon rank-sum test.

**Figure S7** Translation velocity around proline residue in additional dataset for rat, mouse, *E. coli* and human. The vertical dashed line at X = 0 represents the proline residue. The Y-axis represents the average scaled footprints, Y = 1 indicates the baseline of translation velocity (average in each gene). Y > 1 indicates slower translation, while Y < 1 indicates faster translation. Ribbons represent the 95% confidence interval of the mean value.

Figure S7. Translation velocity around proline residue in additional dataset for rat, mouse, *E. coli* and human. The vertical dashed line at X = 0 represents the proline residue. The Y-axis represents the average scaled footprints, Y = 1 indicates the baseline of translation velocity (average in each gene). Y > 1 indicates slower translation, while Y < 1 indicates faster translation. Ribbons represent the 95% confidence interval of the mean value.

**Figure S8** Translation velocity around positively charged amino acid in additional dataset for rat, mouse, *E. coli* and human. The vertical dashed line at X = 0 represents the proline residue. The Y-axis represents the average scaled footprints, Y = 1 indicates the baseline of translation velocity (average in each gene). Y > 1 indicates slower translation, while Y < 1 indicates faster translation. Ribbons represent the 95% confidence interval of the mean value.

Figure S8. Translation velocity around positively charged amino acid in additional dataset for rat, mouse, E. coli and human. The vertical dashed line at X = 0 represents the proline residue. The Y-axis represents the average scaled footprints, Y = 1 indicates the baseline of translation velocity (average in each gene). Y > 1 indicates slower translation, while Y < 1 indicates faster translation. Ribbons represent the 95% confidence interval of the mean value.

**Figure S9** Heatmap of partial spearman correlation coefficients between scaled footprints and 6 features of additional dataset in rat, mouse, *E. coli* and human. The mean of partial correlation coefficients of all the analyzed genes is shown. Bonferroni-corrected P-value for multiple testing were calculated by one sample t-test for the deviation of the mean from zero: **p* < 0.05, ***p* < 2.2e-5, ****p* < 2.2e-10, *****p* < 2.2e-15. N.S, not significant. The raw values of the means and the Bonferroni-corrected P-values are shown in Table S6.

Figure S9. Heatmap of partial spearman correlation coefficients between scaled footprints and 6 features of additional dataset in rat, mouse, E. coli and human. Bonferroni-corrected P-value for multiple testing was used in this study. Statistical significance is indicated as follows: *p < 0.05, **p < 2.2e-5, ***p < 2.2e-10, ****p < 2.2e-15.

**Figure S10** Heatmap of non-partial spearman correlation coefficients between scaled footprints and intrinsically disordered region (IDR) score in N-terminal, Middle and C-terminal in additional dataset for rat, mouse, *E. coli* and human. The mean of partial correlation coefficients of all the analyzed genes is shown. The P-value for multiple testing were calculated by one sample t-test for the deviation of the mean from zero: *p < 0.05, **p < 2.2e-5, ***p < 2.2e-10, ****p < 2.2e-15. N.S, not significant. The raw values of the means and the P-values are shown in Table S7.

Figure S10. Heatmap of non-partial spearman correlation coefficients between scaled footprints and IDR score in N-terminal, Middle and C-terminal in additional dataset for rat, mouse, E. coli and human. Statistical significance is indicated as follows: *p < 0.05, **p < 2.2e-5, ***p < 2.2e-10, ****p < 2.2e-15.
